# Use of Fixed Effects Models to Analyze Self-Controlled Case Series Data in Vaccine Safety Studies

**DOI:** 10.4172/2155-6180.s7-006

**Published:** 2012-04-19

**Authors:** Stanley Xu, Chan Zeng, Sophia Newcomer, Jennifer Nelson, Jason Glanz

**Affiliations:** 1 The Institute for Health Research, Kaiser Permanente Colorado, Denver, CO; 2 School of Public Health, University of Colorado, Aurora, CO; 3 Group Health Research Institute, Seattle, WA

**Keywords:** Self-controlled case series, Adverse events after immunization, Fixed effects model, Longitudinal data, Conditional Poisson model

## Abstract

Conditional Poisson models have been used to analyze vaccine safety data from self-controlled case series (SCCS) design. In this paper, we derived the likelihood function of fixed effects models in analyzing SCCS data and showed that the likelihoods from fixed effects models and conditional Poisson models were proportional. Thus, the maximum likelihood estimates (MLEs) of time-varying variables including vaccination effect from fixed effects model and conditional Poisson model were equal. We performed a simulation study to compare empirical type I errors, means and standard errors of vaccination effect coefficient, and empirical powers among conditional Poisson models, fixed effects models, and generalized estimating equations (GEE), which has been commonly used for analyzing longitudinal data. Simulation study showed that both fixed effect models and conditional Poisson models generated the same estimates and standard errors for time-varying variables while GEE approach produced different results for some data sets. We also analyzed SCCS data from a vaccine safety study examining the association between measles mumps-rubella (MMR) vaccination and idiopathic thrombocytopenic purpura (ITP). In analyzing MMR-ITP data, likelihood-based statistical tests were employed to test the impact of time-invariant variable on vaccination effect. In addition a complex semi-parametric model was fitted by simply treating unique event days as indicator variables in the fixed effects model. We conclude that theoretically fixed effects models provide identical MLEs as conditional Poisson models. Because fixed effect models are likelihood based, they have potentials to address methodological issues in vaccine safety studies such as how to identify optimal risk window and how to analyze SCCS data with misclassification of adverse events

## Introduction

The association between particular adverse events following immunization (AEFI) and receipt of a specific vaccine has been studied using large electronically-linked health care utilization databases [1–8]. As an example, the Vaccine Safety Datalink (VSD) project uses electronic data, including vaccination and diagnosis data, on 8.8 million managed care enrollees to study not only common AEFIs (eg, fever, soreness), but also rare AEFIs (eg, death, seizures, idiopathic thrombocytopenic purpura (ITP)).

Since individuals in observational settings such as the VSD are not randomly chosen to be vaccinated, vaccinated and unvaccinated individuals may differ greatly and possibly in ways related to the outcome of interest. This confounding bias, if not accounted for properly, may invalidate analytic results of cohort studies. In addition, traditional study designs such as matched cohort and case-control designs may not even be feasible for studying vaccine safety because 1) the coverage of some vaccines is nearly 100%, so there are not enough unvaccinated individuals to use for the control group; and 2) data are not collected in safety surveillance system for those who did not experience/report an adverse event (eg, the Vaccine Adverse Event Reporting System). For these reasons, a method known as the self-controlled case series (SCCS) has been developed and widely used for vaccine safety studies [7–11]. The SCCS is a case-only method in which a subject’s follow-up period is partitioned into risk and control intervals. SCCS data are typically analyzed using conditional Poisson models by conditioning on the marginal total number of adverse events that occurred in an individual. The resulting likelihood kernel does not contain the individual-specific random coefficients that explain each individual’s baseline risk for the event count of interest (Farrington, 1995). By making within-person comparisons of incidence rates between vaccine exposed and unexposed time intervals, conditional Poisson models implicitly adjust for all time-invariant individual-level risk factors and potential confounders (measured and not measured).

Several statistical software packages (i.e. STATA, SAS, GENSTAT) can be used for the Poisson process to analyze SCCS data [10,12,13]. Although not explicitly mentioned, fixed effects models were used to analyze SCCS data in a tutorial paper by Whitaker et al. [10]. SCCS data have a longitudinal data structure in which multiple observations are those intervals defined by vaccination exposure status and time-varying covariates on an individual. The number of observations depends on the number of risk levels and levels of time-varying covariates. Fixed effects models have been used for analyzing data with multiple observations on an individual in cohort studies, randomized or observational [14]. They do not assume distributions for the individual-specific random coefficients. The random coefficients are instead estimated from the data as fixed effects in the fixed effects models.

In this paper, we demonstrate that the likelihoods from the fixed effects model and the conditional Poisson model are proportional when analyzing SCCS data. We show that by simulation, fixed effects models and conditional Poisson models typically used for SCCS data analysis are equivalent in estimating vaccination effects. We also compared these two approaches to a generalized estimating equation approach (GEE) [15,16], which is widely used for longitudinal data analysis. We furthermore demonstrate that fixed effects models have numerous advantages over the conditional Poisson models when analyzing a SCCS example.

## Methods

### Conditional Poisson models for self-controlled case series design

If a Poisson process is assumed for the unrestricted population in a cohort design, the likelihood function, conditioning on each individual’s total number of adverse events is a multinomial kernel, containing only the parameters βs for time-varying covariates including the exposure effect β_1_. Since the individual effects are canceled out in this analysis, it is not to be expected that any assumption of their distribution would influence the inference of βs. The multinomial term is zero for all individuals with no adverse event. Thus, individuals without adverse events do not contribute in parameter estimation.

Suppose we have a sample of *n* individuals with adverse events. Let R = exp(β_1_) denote the incidence rate ratio for the vaccination effect where β_1_ is the model coefficient for the vaccination effect. Farrington (1995) proposed a conditional Poisson model that uses only cases, vaccinated and unvaccinated. The conditional Poisson likelihood kernel is the product of the likelihood kernel across subjects which is of the following form for the *i*^th^ subject

(1) $$L_{cp(i)}=\prod_{j}{\left\{\frac{t_{ij}\exp({\text{X}}_{ij}\beta )}{\sum_{j}t_{ij}\exp({\text{X}}_{ij}\beta )}\right\}}^{y_{ij}}$$

Here, X_ij_ is the row vector of time-varying covariates including indicator variables for age effects and vaccination effect, β is a column vector of corresponding coefficients including β_1_, t_ij_ is the person-time (in days) for subject *i* in interval *j*, and y_ij_ is the corresponding number of adverse events for subject *i* in interval *j* which is binary when the adverse event is rare. The conditional Poisson regression model (1) allows for more than one risk levels in the risk window [10].

### Fixed effects models for SCCS data: parametric and semi-parametric models

SCCS data have longitudinal data structures where analytic units are intervals of each subject. Each interval represents a unique combination of time-varying covariates. Although each individual has at least one adverse event, some intervals of an individual do not have an adverse event. In this paper it is assumed that the observations in an SCCS data set follow a Poisson distribution in which the individual effects may be represented by a random coefficient. As a consequence, the likelihood function from the fixed effects model is the full likelihood function for SCCS data. Let ξ_i_ represent the individual-specific random coefficient for individual i who experienced at least one adverse event during the follow-up period T_i_, and ξ_i_ does not assume any defined distribution such as a normal distribution.

We consider fitting the fixed effects models for SCCS data,

(2) $${\text{y}}_{\text{ij}}\sim \text{Poisson}\;({\text{u}}_{\text{ij}})$$

where u_ij_=t_ij_ m_i_ λ_ij_, m_i_=exp(α+ξ_i_) and λ_ij_ =exp(X_ij_**β**), α represents the intercept coefficient for the overall case sample, ξ_i_ is the unknown individual-specific random effect, X_ij_ and β have the same meanings as defined previously for model (1), and exp(α) is the baseline incidence rate for the adverse events for each unit of unvaccinated period of time (eg, each day) in the case sample. Similar to the likelihood function for cohort data [14], the likelihood function of the fixed effects model for individual *i* when using case-only data is,

$$L_{FE(i)}=\prod_{j=1}^{J_{i}}\frac{\exp(u_{ij})u_{ij}^{y_{ij}}}{y_{ij}!}$$

where J_i_ is the number of observations on individual *i*. Substituting u_ij_= t_ij_ m_i_ λ_ij_,

$$\begin{array}{l} L_{FE(i)}=\prod_{j=1}^{J_{i}}\frac{\exp({\text{t}}_{\text{ij}}m_{i}\lambda_{ij}){\left({\text{t}}_{\text{ij}}m_{i}\lambda_{ij}\right)}^{y_{ij}}}{y_{ij}!}\\ =\left(\prod_{j}\frac{1}{y_{ij}!}\right)\exp\left(m_{i}\sum_{j}t_{ij}\lambda_{ij}\right)\prod_{j}{\left(m_{i}t_{ij}\lambda_{ij}\right)}^{y_{ij}} \end{array}$$

Taking the natural logarithm of *L_FE_*_(_*_i_*_)_, differentiating with respect to *m_i_* and setting to zero we have,

$${\overset{⌢}{m}}_{i}=\frac{\sum_{j}y_{ij}}{\sum_{j}t_{ij}\lambda_{ij}}$$

Substituting into *L_FE_*_(_*_i_*_)_, the likelihood function for the *i*^th^ individual is

$$\begin{array}{l} L_{FE(i)}=\left(\prod_{j}\frac{1}{y_{ij}!}\right)\exp\left(\sum_{j}y_{ij}\right)\prod_{j}{\left(t_{ij}\lambda_{ij}\left(\frac{\sum_{j}y_{ij}}{\sum_{j}t_{ij}\lambda_{ij}}\right)\right)}^{y_{ij}}\\ =\left(\prod_{j}\frac{1}{y_{ij}!}\right)\exp\left(\sum_{j}y_{ij}\right)\prod_{j}{\left(\sum_{j}y_{ij}\right)}^{y_{ij}}\prod_{j}{\left(\frac{t_{ij}\lambda_{ij}}{\sum_{j}t_{ij}\lambda_{ij}}\right)}^{y_{ij}}\propto \prod_{j}{\left(\frac{t_{ij}\lambda_{ij}}{\sum_{j}t_{ij}\lambda_{ij}}\right)}^{y_{ij}} \end{array}$$

Substitute λ_ij_ = exp(**X***_ij_***β**) into *L_FE_*_(_*_i_*_)_

(3) $$L_{FE(i)}\propto \prod_{j}{\left(\frac{t_{ij}\exp({\text{X}}_{ij}\beta )}{\sum_{j}t_{ij}\exp({\text{X}}_{ij}\beta )}\right)}^{y_{ij}}=L_{CP(i)}$$

We show that the likelihood function from a fixed effects model as in (3) is proportional to that from a conditional Poisson model as in (1). As a result, the maximum likelihood estimates of βs are same for the two models.

Age is an important confounder in vaccine safety studies because of its possible association with both the probability of vaccination and the occurrence of adverse events. A model with a defined age effect form such as the age groups used in the MMR-ITP example is called a parametric model while a model without defining age effect form is called a semi-parametric model [10,12]. Fixed effects models as in (3) can be easily modified to fit the semi-parametric model in which the form of age effects is not specified. Each event age is treated as a dummy variable, so essentially the length of an age group is only one day, and the number of age effect levels is the number of unique days of adverse events.

## Results

### Simulation study and results

We performed simulations to evaluate the performance of a fixed effects model in its ability to analyze SCCS data. We also compared estimates from a fixed effects model and conditional Poisson model to those obtained using generalized estimating equations (GEE) [15,16], which has been widely used to analyze longitudinal data including normally distributed data, binary and Poisson data. GEE is not a likelihood-based approach and is used to estimate population average parameters and correlation among observations on the same individual is accounted for.

#### Simulation

Dependent Poisson data were simulated according to model (2) with ξ_i_ independent and identically distributed N (0, σ^2^) or a uniform distribution (0, 1). σ was chosen to be 1 and 1.5. Each of 100 individuals was assumed to have a follow-up period of 365 days, consisting of both risk and control periods. Typically, there are three periods for each subject: the control period before vaccination, the risk period after vaccination, and the control period after the risk period. Vaccination times were assumed to follow a normal distribution with a mean of 140 days and a standard deviation of 42 days. The values of the overall intercept coefficient, α, were set to be −5, −6, −7, and −8 to achieve baseline incidence rates ranging from 0.00034 to 0.00674 per day in the simulated data. Time-varying covariates included in the model were age effects and an indicator variable for risk and control periods. The chosen coefficients for age effects were 0.3 for days 91–270 and 0.1 for days 271–365 with days 1–90 as the reference group. The coefficient for vaccination effect, β_1_, was chosen to be 0.69, 1.39, and 1.79, which represents incidence rate ratios of 2, 4 and 6, respectively. 1,000 datasets were simulated and analyzed for each combination of parameters.

#### Evaluation measures

We analyzed each simulated dataset with three methods, a conditional Poisson model, a fixed effects model and GEE. Means of vaccination effects and their standard errors for each of the three methods in each simulated data setting were calculated. We also calculated the means of absolute difference between conditional Poisson models and GEE, and reported the maximum absolute difference. We conducted this last step because it is possible that the means of the vaccination effect estimates are same between the conditional Poisson models and GEE, but vaccination effect estimates may differ in each simulated data set. Empirical powers were calculated as percent of data sets with significant vaccination effect (p-value<0.05) under the alternative hypothesis. Type I error rates were also reported for data simulated under the null hypothesis, β_1_=0.

#### Simulation results

Table 1 shows that conditional Poisson models, fixed effects models, and GEE have acceptable type I error rates (i.e. about 5%) for the parameters examined when the individual-specific random errors are simulated from a normal distribution. The GEE approach produces slightly different estimates and their standard errors for vaccination effect than conditional Poisson models and fixed effects models (Table 2). Empirical powers are also similar among the three approaches under the alternative hypothesis β_1_ ≠ 0. Although there is little difference on average in parameter estimation between GEE and conditional Poisson models, the means of absolute difference between conditional Poisson models and GEE reveal that they can produce very different results for some simulated data sets. For example, the maximum absolute difference in estimated coefficient for the vaccine effects is 0.339 in a simulated data set while the mean of the absolute difference is only 0.0127 (Table 3). Similar results are observed for β_1_=1.39 and 1.79 (data not shown). When the individual-specific random errors are simulated from a uniform distribution, similar results are observed as well (data not shown).

### An example

To demonstrate how a fixed effects model approach can be used for SCCS data, we re-analyzed the data from a SCCS study examining the association between MMR vaccination and ITP among a US pediatric population [7]. They excluded the 42-day healthy period immediately preceding MMR vaccination and used a pre-specified 42-day risk window after MMR vaccination, which has subsequently been confirmed by a data-driven approach to be an optimal choice of risk window length [17]. They found an incidence rate ratio of 7.06 for those vaccinated at an age between 366 and 690 days old. In this paper, for the purpose of demonstrating the use of fixed effects models in analyzing SCCS data, we defined six age groups as in Xu et al. [17]: 366–426 days, 427–487 days, 488–548 days, 549–609 days, 610–670 days, and 671–730 days (the reference group). Days outside of a 42-day post-vaccination risk window were considered to be the control window. We also included all subjects with follow-up of 366–730 days regardless of vaccination status and without excluding the healthy period before MMR vaccination.

The first step in applying a fixed effects model to SCCS data involves manipulating the data to a more usable format. Specifically, the analytic data can be expanded as described in Xu et al. [13]. Briefly, the follow-up period for each individual in the SCCS sample was expanded into daily observations with an exposure status indicator (risk versus control), indicators for age effects (defined age effects group for parametric model or undefined age effects form for semi-parametric model), an indicator variable for adverse events, and any other time-invariant and time-varying covariates. Data in this expanded form facilitate simple calculations of the person time (in days) and number of adverse events by exposure status, age groups, and any other time-varying covariates for each individual. The result is a data set with multiple observations for each individual, which can be analyzed using a fixed effects model with most statistical software packages (eg, PROC GENMOD in SAS).

Table 4 shows that conditional Poisson models and fixed effects models give the same estimates and standard errors for both the vaccination effect and the age effects when using the parametric method for age effects. Estimates and standard errors obtained using a GEE approach differ only slightly. For the semi-parametric method, the conditional Poisson model and fixed effects model yield the same estimates for the vaccination effect (1.97) and standard error (0.37). Estimates using GEE are not available when using the semi-parametric model because the individual-specific coefficients cannot be modeled as both fixed and random effects in the same model.

To show one of many potential utilities of using a fixed effects model for SCCS data, we also compared the risk of ITP after MMR vaccination between male and female patients. If conditional Poisson models are employed, gender subgroup analyses must be carried out to make this comparison, and a direct statistical comparison (test) is not readily available. However, if a fixed effects model is used, we can statistically compare the risk of ITP between genders by fitting a model with two interactions 1) between exposure and gender and 2) between age groups and gender (Table 5). The test statistic and p-value for this comparison is readily available in results of standard statistical software (eg, using estimate and contrast statements in PROC GENMOD in SAS). Additionally, the fixed effects model can accommodate the same age effects for both male and female patients by replacing the interaction between age groups and gender with age groups only (Table 5). This model produced smaller gender difference in risks of ITP after MMR vaccination than the model with different age coefficients for male and female patients although the conclusion is same (p-value = 0.76 versus 0.31). In this way, the fixed effects modeling approach offers considerable additional flexibility beyond what a conditional Poisson approach can provide.

## Discussion

Fixed effects models were first implicitly recommended for analyzing SCCS data by Whitaker et al. [10]. They included a factor for each individual to ensure the fitted individual totals equal the observed values but did not study the relation between the conditional Poisson models and fixed effects models. In this paper, we demonstrate that theoretically and by simulation the parameters estimates and their standard errors are equivalent between the conditional Poisson models and fixed effects models. On average, GEE approach produces very similar estimates and their standard errors for vaccination effects although for some simulated data sets it can produce very different results than the conditional Poisson models. We do not recommend using GEE approach to analyze SCCS data due to possible bias in estimation.

There are numerous of advantages for statistical analysts in vaccine safety research to use fixed effects models to analyze SCCS data. First, likelihood-based statistical tests may be employed. For example, a likelihood ratio test was used to investigate the effect of gender on the association between oral polio vaccine and intussusception [10]. Second, statistical analysts may find fitting fixed effects models for SCCS data more intuitive than fitting conditional Poisson models in standard statistical software packages because they are already familiar with procedures for fitting Poisson models (eg, PROC GENMOD in SAS). Third, fitting a complex semi-parametric model is achieved by simply treating event days as indicator variables in preparing summary data for person time and number of events and later including them in the fixed effects model.

Although we examined a wide variety of simulated data settings, we only studied normal and uniform distributions for the individual-specific random effects in simulations. Results should remain equivalent for conditional Poisson models and fixed effects models for different distributions since their likelihoods are proportional. Inconsistent estimation may be a potential issue when fitting fixed effects models if the number of random effects approaches infinity. However, in SCCS vaccine safety studies, the number of random effects to be estimated depends on the number of individuals with adverse events, which is usually small. Thus, inconsistency should not often be a problem when analyzing SCCS data. For relatively large number of cases, the fixed effects models may take relatively long time to fit. In this case Whitaker et al. [10] suggested the use of absorbing factors to fit the models efficiently without estimating individual-specific random effects explicitly.

In summary, for vaccine safety studies involving SCCS data, we recommend using a fixed effects modeling framework to estimate incidence rate ratios as this approach offers many advantages compared to the traditionally-used conditional Poisson model. More broadly, existing longitudinal data analysis tools may provide opportunities to better address research questions that arise in SCCS vaccination studies such as how to use likelihood-based statistical method to identify optimal risk windows for a given SCCS data set [17] how to analyze SCCS data with misclassification of adverse events when adverse events are partially chart reviewed due to resource limitation [18–20].

## Figures and Tables

**Table 1:** Type I error rates from 1000 simulations under the null hypothesis, β_1_=0, when individual-specific random coefficients have a normal distribution.

Simulation parameters		Average number of cases	Type I error rates (%)	
α	σ		CP^a^/FE^b^	GEE^c^
−5	1	488	4.0	4.3
	1.5	896	4.3	4.0
−6	1	179	3.7	4.0
	1.5	336	5.0	5.1
−7	1	66	4.4	4.3
	1.5	124	2.8	2.5
−8	1	25	6.2	6.0
	1.5	46	4.1	4.0

a CP, conditional Poisson model.

b FE, fixed effects model.

c GEE, generalized estimation equations.

**Table 2:** Mean of vaccination effect coefficient (standard error) and empirical power from 1000 simulations with true β_1_=0.693 when individual-specific have a normal distribution.

Simulation parameters		Average number of cases	Mean of vaccination effect coefficient (standard error)		Empirical power (%)	
α	σ		CP^a^/FE^b^	GEE^c^	CP/FE	GEE
−5	1	510	0.682 (0.159)	0.687 (0.159)	96	97
	1.5	936	0.664 (0.127)	0.662 (0.132)	100	100
−6	1	188	0.668 (0.279)	0.668 (0.280)	68	68
	1.5	351	0.665 (0.207)	0.661 (0.209)	87	87
−7	1	69	0.600 (0.516)	0.599 (0.516)	35	35
	1.5	130	0.661 (0.355)	0.655 (0.356)	55	54
−8	1	26	0.719 (0.624)	0.718 (0.624)	17	17
	1.5	48	0.618 (0.574)	0.611 (0.578)	26	26

a CP, conditional Poisson model.

b FE, fixed effects model.

c GEE, generalized estimation equations.

**Table 3:** Mean of absolute difference of vaccination effect coefficient from 1000 simulations with true β_1_=0.693 when individual-specific random effects have a normal distribution.

Simulation parameters	Mean of absolute difference (maximum)	
α	σ	CP^a^/FE^b^ versus GEE^c^
−5	1	0.0081 (0.0671)
	1.5	0.0086 (0.0873)
−6	1	0.0064 (0.1576)
	1.5	0.0080 (0.1931)
−7	1	0.0060 (0.1175)
	1.5	0.0099 (0.2884)
−8	1	0.0068 (0.2174)
	1.5	0.0127 (0.3393)

a CP, conditional Poisson model.

b FE, fixed effects model.

c GEE, generalized estimation equations.

**Table 4:** Estimated coefficients (standard error) using parametric method from US MMR-ITP data.

	CP^a^/FE^b^	GEE^c^
Vaccination	2.00 (0.36)	2.04 (0.38)
366–426	−1.50 (0.55)	−1.55 (0.61)
Age (days)		
427–487	−0.39 (0.44)	−0.39 (0.42)
488–548	−0.30 (0.42)	−0.31 (0.44)
549–609	−0.27 (0.42)	−0.31 (0.43)
610–670	−0.14 (0.41)	−0.16 (0.42)

a CP, conditional Poisson model.

b FE, fixed effects model.

c GEE, generalized estimation equations.

**Table 5:** Estimated coefficients (standard error) by gender using parametric method from US MMR-ITP data.

	Subgroup CP^a^/FE^b^ with gender*age and exposure*gender		FE with exposure*gender and age	
	Female	Male	Female	Male
Vaccination	2.64 (0.64)	1.85 (0.45)	1.90 (0.51)	2.01 (0.44)
Age (days)				
366–426	−3.11 (1.00)	−0.42 (0.75)	−1.49 (0.56)	
427–487	−0.84 (0.60)	0.15 (0.68)	−0.39 (0.44)	
488–548	−1.37 (0.66)	0.62 (0.63)	−0.28 (0.42)	
549–609	−0.40 (0.53)	−0.06 (0.69)	−0.28 (0.42)	
610–670	−0.58 (0.57)	−0.43 (0.64)	−0.14 (0.41)	

a CP, conditional Poisson model.

b FE, fixed effects model.

## References

[1] Black S, Shinefield H, Ray P, Lewis E, Chen R (1997). Risk of hospitalization because of aseptic meningitis after measles-mumps-rubella vaccination in one-to two-year-old children: an analysis of the Vaccine Safety Datalink (VSD) project. Pediatr Infect Dis J.

[2] Black C, Kaye JA, Jick H (2003). MMR vaccine and idiopathic thrombocytopenic purpura. Br J Clin Pharmacol.

[3] Chen RT, Glasser JW, Rhodes PH, Davis RL, Barlow WE (1997). The Vaccine Safety Datalink Project: A new tool for improving vaccine safety monitoring in the United States. Pediatrics.

[4] Chen RT, Plotkin SA, Orenstein WA (1999). Safety of vaccines. Vaccines.

[5] DeStefano F, Mullooly JP, Okoro CA, Chen RT, Marcy SM (2001). Childhood vaccinations, vaccination timing, and the risk of type I diabetes mellitus. Pediatrics.

[6] France EK, Glanz JM, Xu S, Davis RL, Black SB (2004). Safety of the trivalent inactivated influenza vaccine among children: A population-based study. Arch Pediatr Adolesc Med.

[7] France EK, Glanz J, Xu S, Hambidge S, Yamasaki K (2008). Risk of immune thrombocytopenic purpura after measles-mumps-rubella immunization in children. Pediatrics.

[8] Hambidge SJ, Glanz JM, France EK, McClure D, Xu S (2006). Safety of trivalent inactivated influenza vaccine in children 6 to 23 months old. JAMA.

[9] Farrington CP (1995). Relative incidence estimation from case series for vaccine safety evaluation. Biometrics.

[10] Whitaker HJ, Farrington CP, Spiessens B, Musonda P (2006). Tutorial in Biostatistics: The self-controlled case series method. Stat Med.

[11] Whitaker HJ, Hocine MN, Farrington CP (2009). The methodology of self-controlled case series studies. Stat Methods Med Res.

[12] Farrington CP, Whitaker HJ (2006). Semiparametric analysis of case series data (with discussion). Applied Statistics.

[13] Xu S, Gargiullo P, Mullooly J, McClure D, Hambidge S (2010). Fitting parametric and semi-parametric conditional Poisson regression models with Cox’s partial likelihood in self-controlled case series and matched cohort studies. Journal of Data Science.

[14] Cameron AC, Trivedi PK (1998). Regression Analysis of Count Data.

[15] Liang KY, Zeger SL (1986). Longitudinal data analysis using generalized linear models. Biometrika.

[16] Zeger SL, Liang KY (1986). Longitudinal data analysis for discrete and continuous outcomes. Biometrics.

[17] Xu S, Zhang L, Nelson JC, Zeng C, Mullooly J (2011). Identifying optimal risk windows for self-controlled case series studies of vaccine safety. Stat Med.

[18] Whittemore AS, Gong G (1991). Poisson regression with misclassified counts: application to cervical cancer. J R Stat Soc Ser C Appl Stat.

[19] Mullooly JP (1996). Misclassification model for person-time analysis of automated medical care databases. Am J Epidemiol.

[20] Mullooly JP, Donahue JG, DeStefano F, Baggs J, Eriksen E (2008). Predictive Value of ICD-9-CM Codes Used in Vaccine Safety Research. Methods Inf Med.

